# A Biorefinery Approach to the Biomass of the Seaweed *Undaria pinnatifida* (Harvey Suringar, 1873): Obtaining Phlorotannins-Enriched Extracts for Wound Healing

**DOI:** 10.3390/biom11030461

**Published:** 2021-03-19

**Authors:** Carolina A. M. Ferreira, Rafael Félix, Carina Félix, Adriana P. Januário, Nuno Alves, Sara C. Novais, Juliana R. Dias, Marco F. L. Lemos

**Affiliations:** 1 MARE—Marine and Environmental Sciences Centre, ESTM, Politécnico de Leiria, 2050-641 Peniche, Portugal; carolina.ferreira@ipleiria.pt (C.A.M.F.); rafael.felix@ipleiria.pt (R.F.); carina.r.felix@ipleiria.pt (C.F.); adriana.p.januario@ipleiria.pt (A.P.J.); sara.novais@ipleiria.pt (S.C.N.); 2 CDRSP—Centre for Rapid and Sustainable Product Development, Politécnico de Leiria, 2030-028 Marinha Grande, Portugal; nuno.alves@ipleiria.pt (N.A.); juliana.dias@ipleiria.pt (J.R.D.)

**Keywords:** anti-inflammatory, antimicrobial, antioxidant, circular economy, phlorotannins

## Abstract

Brown seaweeds are recognized sources of compounds with a wide range of properties and applications. Within these compounds, phlorotannins are known to possess several bioactivities (e.g., antioxidant, anti-inflammatory, and antimicrobial) with potential to improve wound healing. To obtain phlorotannins enriched extracts from *Undaria pinnatifida*, a biorefinery was set using low-cost industry-friendly methodologies, such as sequential solid–liquid extraction and liquid–liquid extraction. The obtained extracts were screened for their antioxidant and antimicrobial activity against five common wound pathogens and for their anti-inflammatory potential. The ethanolic wash fraction (wE100) had the highest antioxidant activity (114.61 ± 10.04 mmol·mg^−1^ extract by Diphenyl-1-picrylhydrazyl (DPPH) and 6.56 ± 1.13 mM eq. Fe II·mg^−1^ extract by and Ferric Reducing Antioxidant Power (FRAP)), acting efficiently against Gram-negative (*Pseudomonas aeruginosa*) and Gram-positive (*Staphylococcus aureus*) bacteria, and showing a nitric oxide production inhibition over 47% when used at 0.01 µg·mL^−1^. NMR and FTIR chemical characterization suggested that phlorotannins are present. Obtained fraction wE100 proved to be a promising candidate for further inclusion as wound healing agents, while the remaining fractions analyzed are potential sources for other biotechnological applications, giving emphasis to a biorefinery and circular economy framework to add value to this seaweed and the industry.

## 1. Introduction

The biodiversity that characterizes the marine environment has contributed to the bioprospection of unique compounds in the last years, culminating in an unprecedented development of blue biotechnology [1]. A major part of the research in this field has focused on seaweeds, as they are recognized potential sources of biotechnologically relevant extracts and/or compounds for a myriad of applications [2]. In fact, brown seaweeds are the most economically valuable due to the application of their bioactive compounds, namely alginate, in the food and beverage industry, with a market valued at USD 706.9 million in 2019 [3]. Alginate is also widely used in the pharmaceutical industry (wound care) due to its biocompatibility, being used in trademarks such as ALGICELL (Dermasciences, Torrance, CA, USA) and ALGISITE (Smith & Nephew, Hull, UK) [4]. Other valuable bioactive compounds in brown seaweeds are sulphated polysaccharides, carotenoids, polyphenols, and phytosterols [5,6]. The polyphenolic compounds of brown seaweed are a group of molecules exclusively produced by these organisms, known as phlorotannins. Phlorotannins are composed by phloroglucinol (1,3,5-tryhydroxybenzene) polymers, which can be grouped into four classes: fuhalols and phlorethols, with ether linkages (Aryl-O-Aryl); fucols with phenyl linkages (Aryl-Aryl); fucophlorethols, which have both ether and phenyl linkages and can be branched; phlorotannins with dibenzodioxin linkages—eckols and carmalols [7]. Depending on their molecular size and polymerization degree, their functions and activities can vary. Phlorotannins have been widely investigated for their antidiabetic, antimicrobial, antiallergic, and anti-inflammatory activities, among others [8], and may thus be potential bioactive additives in wound healing treatment [9].

*Undaria pinnatifida* (Harvey Suringar, 1873) is an invasive brown seaweed, originally from Northeast Asia (Japan, Korea, and China), that was detected in the Atlantic Ocean, specifically in the north of Portugal, in 1999, and has progressed along the entire coast [10,11,12,13]. This invasive species competes with native algal communities, which can lead to ecological and economic problems. However, this seaweed shows several bioactivities with biotechnological potential, as antioxidant, anti-inflammatory, antitumor, antihypertensive, antiviral, and antiobesity properties [13], which can represent an opportunity if duly valued, namely in the advanced wound care market [14,15]. Thus, the utilization of this biomass under a biotechnological valorization platform could be of double benefit, serving both a bioeconomic growth and a localized ecosystem remediation by prompting harvesting efforts of this species. However, under the regulation No 1143/2014 of the European Parliament, invasive alien species (IAS) cannot be intentionally marketed, despite not being clear if that is the case for already established species for which a market was created after invasion, with the purpose of promoting population control and damage mitigation. Actually, Pasko et al. [16] state that among conservationists, the promotion of a “harvest incentive strategy” is of utmost importance for IAS control, and among such incentives there is the development of a commercial market where the species is valorized. As such, this is a topic that should be carefully addressed, as turning threats into opportunities [15] can be a great asset if done ethically and with the best practices.

The wound healing process is complex and requires a sequential cascade of events, where nitric oxide (NO) plays an important role in antimicrobial activities, vasodilation, inflammation response, cell proliferation, angiogenesis promotion, and matrix deposition, culminating in the reorganization of the injury [17]. However, in excessive concentrations, NO is toxic and originates oxidative stress, which causes cellular death and avoids reepithelization [18,19]. Phlorotannins, specifically eckols and phloroeckols, have already been shown to suppress the activity of proinflammatory enzyme’s expression, such as inducible NO synthase (iNOS) and cyclooxygenase-2 (COX-2), which are responsible for the large production of NO [20]. Other problems associated with wounds are the infections caused by bacterial organisms (such as *Staphylococcus aureus*, *Pseudomonas aeruginosa*, or *Escherichia coli*). Effective treatment against wounds’ bacterial infection remains a challenge, which has been further augmented by the development of antibiotic resistance by some pathogens, and new wound-healing products that possess antimicrobial effects are extremely required.

Extracting specific compounds from seaweed is a laborious task with several costs associated, particularly for phlorotannins, since these compounds are usually linked with proteins and polysaccharides and are easily coextracted with much more abundant sugars (mannitol) [21]. The solvent choice is a critical step and protocols for polyphenols extraction are widely variable, being water, ethanol, and mixtures of both among the most used [7,22], but other less polar mixtures are used as well such as 70:30 acetone:water [7]. Ethyl acetate has generally been used to concentrate the extracts in phenolic compounds, resulting in the highest results for antioxidant activity [7,23].

Despite significant advances in novel extraction techniques for natural compounds, the majority have very high costs when used at the industrial scale. For this reason, the traditional methods are preferred, such as solid–liquid extraction (SLE) and liquid–liquid extraction (LLE). However, these processes usually generate leftovers and waste products that are disposed of, and in a world where global economies have been pressured to implement sustainable measures, new alternatives to avoid this scenario are crucial [24]. Using a sequential extraction, the biomass may be continuously extracted by the utilization of several solvents with different polarities following the expected target. This approach increases the resource efficiency and adds an even higher value to the used biomass, certifying a circular economy concept, with sustainable use of resources, while feeding different industrial sectors [25].

In the present study, several added-value extracts, among which a phlorotannins-enriched extract, were obtained from *U. pinnatifida* biomass using low-cost, sequential extraction and fractionation approaches, and posteriorly characterized for their further application in biomedicine, namely for wound healing.

## 2. Materials and Methods

### 2.1. Biomass Harvesting and Processing

*Undaria pinnatifida* (Heterokontophyta: Phaeophyceae) was collected from Estai Cape (Longitude: O8°48′53.35″, Latitude: N42°11′13.24″) in Spain. After collection, the fresh algal biomass was sorted and sediments and epibionts removed, rinsed with freshwater, and dried in a wind tunnel at 25 °C, and after, ground to a fine flour-like powder (50 μm). This powder was then stored under vacuum, in the dark, at room temperature until usage.

### 2.2. Preliminary Sequential Extraction Screening

To recover a fraction of *U. pinnatifida* enriched in phlorotannins (less complex than a single-step crude extract), while promoting a circular economy approach to the fractions thereby obtained, a preliminary series of sequential solid–liquid extractions (SLE) and liquid–liquid extractions (LLE) was designed and performed (a comprehensive flowchart is presented in Supplementary Materials (S1)). The powdered biomass (20 g) was soaked in 300 mL of ice-cold *n*-hexane (solid-to-liquid ratio 1:15), and agitated on a magnetic stirrer for 20 min, in the dark to avoid oxidative degradation of compounds, and on an ice bath to prevent temperature increase [7]. To maximize the extraction efficacy, after 20 min, the liquid extract was removed by centrifugation (5 min at 5000× *g* at 4 °C), 300mL of fresh solvent was added to the recovered leftover biomass and this was re-extracted for an additional 20 min. The procedure was then repeated a third time (for each solvent, three extractions were performed). The extracts recovered in each of the three washes of a given solvent were pooled and stored at 4 °C, resulting in the “hexane extract” (H). The extraction continued with water:acetic acid (99:1) (AQ), maintaining the previous extraction conditions: 300 mL per wash, 20 min, magnetic stirring, ice bath, darkness, and three washes with fresh solvent of the same type. Between different solvents, instead of centrifugation, biomass was recovered by filtration (Whatmann filter n° 1, Cytiva, UK). After AQ, the extraction followed with ethanol:water:acetic acid (50:49.5:0.5) (E50), ethanol (E100), ethanol:acetone (1:1) (A50), acetone (A100), and ethyl acetate (AE).

The extracts were semipurified to separate the different classes of compounds according to solvent affinity. The extract H (hexane) was washed with methanol. As methanol and hexane are partially miscible, a few drops of water were added to promote phase separation. Thus, the fraction hexane-methanol (HM), and the leftover fraction “washed hexane” (wH) were produced. The aqueous extract (AQ) was partitioned into three fractions. First, the liquid extract was fractionated with hexane, forming the aqueous-hexane fraction (AQH), and then it was extracted with ethyl acetate (AQAE), once again recovering the leftover fraction (wAQ). The aqueous ethanol extract (E50) was extracted with hexane, forming the aqueous ethanol-hexane fraction (E50H) and the leftover fraction (wE50). Following the same rationale, E100, A50, and A100 were each washed with hexane, forming E100H and wE100, A50H and wA50, and A100H and wA100. The last treatment was the wash with water:acetic acid (99:1) in the ethyl acetate (EA) extract, resulting in ethyl acetate-aqueous fraction (AEAQ), and the leftover, wEA.

After LLE, all 15 fractions were evaporated to dryness in a rotary evaporator under vacuum and a water bath (maximum temperature of 45 °C), and yields were calculated based on the dry weight. Next, all fractions were resuspended in dimethyl sulfoxide (DMSO, Carlo Erba, Spain), to obtain stock samples with concentrations between 10 and 100 mg·mL^−1^, depending on sample’s solubility. Extracts from this preliminary sequential extraction were tested for DPPH and FRAP antioxidant activities as well as antibacterial activity against *Staphylococcus aureus* by the disc diffusion method (methods in following sections).

### 2.3. Optimized Sequential Extraction

**Figure 1:**
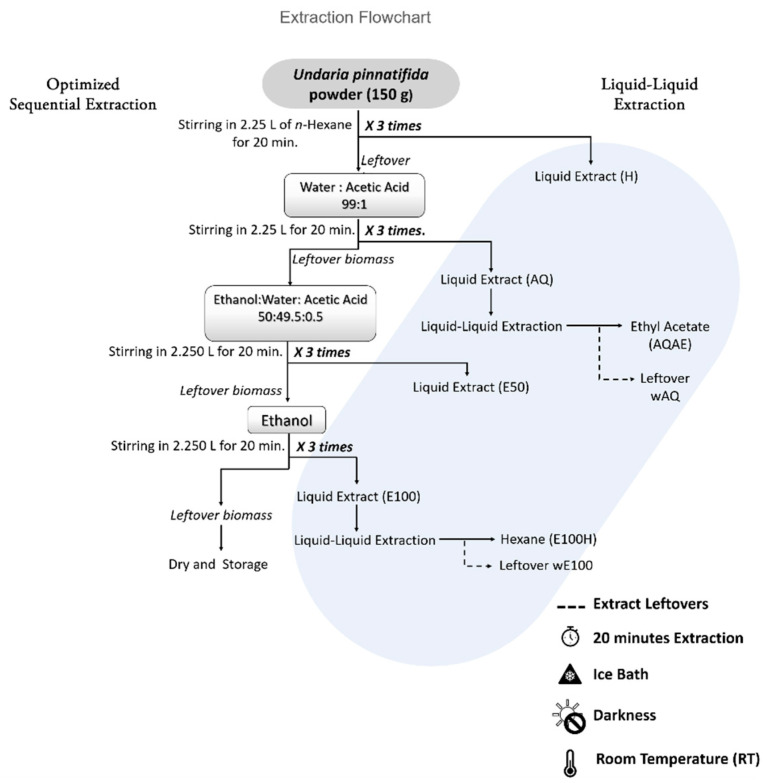
Scheme of the optimized sequential extraction of *Undaria pinnatifida* and the liquid–liquid extraction with the respective outputs (final six extracts on shadowed region).

Depending on the results of Section 2.2 (preliminary sequential extraction), mostly yield and utility of LLE (see S1), several steps of the preliminary design were discarded, and an optimized sequential extraction was performed at a larger scale to produce higher amounts of target extracts (see Figure 1). Thus, 150 g of *U. pinnatifida* biomass were extracted under the following conditions: three washes per solvent, 20 min per wash, darkness, ice bath, and magnetic stirring. Separation of extract and biomass was maintained as before: centrifugation between washes of the same solvent and filtration between different solvents. The solvent pipeline was as follows: hexane > water:acetic acid (99:1) > ethanol:water (1:1) > and ethanol.

After, AQ and E100 crude extracts were semipurified through liquid–liquid extraction. The AQ extract was washed with ethyl acetate, originating the fractions AQAE and wAQ. Additionally, E100 extract was separated with hexane, forming E100H, and wE100 fractions (Figure 1). Before analysis, the extracts and fractions were evaporated to dryness in a rotary evaporator, dissolved in DMSO, and stored in aliquots for future chemical analysis under the previously mentioned conditions.

### 2.4. Antioxidant Activity

#### 2.4.1. Diphenyl-1-picrylhydrazyl (DPPH) Assay

This assay was performed according to Félix et al. [26], with minor modifications, preparing the working reagent by dissolving DPPH (Sigma Aldrich, St. Louis, MO, USA) radical in absolute ethanol at a concentration of 0.1 mg·mL^−1^. Briefly, the extracts previously prepared in DMSO varying concentrations between 10 and 100 mg·mL^−1^ were used. In a 96-well microplate, 10 μL of each extract/standard concentration and DMSO as control, were pipetted per well (eight wells each). In four wells, 190 μL of working reagent was added, and in the other four, 190 μL of ethanol was added. The plate was incubated in the dark for 60 min at room temperature, and after this period, the absorbance was read at 515 nm (EPOCH 2 microplate reader, BioTek^®^ Instruments, Winooski, VT, USA). The amount of DPPH radical reduced was calculated using a standard curve previously obtained, and by subtracting the mean absorbance of the wells containing sample and ethanol to the mean absorbance of the wells containing sample and working reagent. Three independent assays were performed, and the results expressed as millimole per milligram of seaweed extract (mmol·mg^−1^ extract).

#### 2.4.2. Ferric Reducing Antioxidant Power (FRAP) Assay

This method is based on the reduction, at low pH, of a colorless ferric complex (Fe^3+^ tripyridyltriazine) to a blue-colored ferrous complex (Fe^2+^ tripyridyltriazine) by the action of electron-donating antioxidants. The reduction is monitored by measuring the change of absorbance at 593 nm [27]. The FRAP working reagent (WR) was prepared daily by mixing 10 volumes of 300 mM acetate buffer, (pH 3.6), to 1 part of 10 mM TPTZ (2,4,6-tri(2-pyridyl)-s-triazine, Sigma Aldrich, St. Louis, MO, USA) in HCl 40 mM and 1 part of 20 mM FeCl_2_ (ferric chloride). The assay was conducted by pipetting 8 µL of each standard/sample’s concentration per well, or 8 µL of DMSO as control (eight wells/condition). In four of the eight wells, 272 µL of WR was added, and in the other four wells, 272 µL of water was added (blanks). Additionally, four wells containing 8 µL of FeSO_4_ (1 mM) and 272 µL of WR (positive control) and four wells containing 280 µL of water were added. The plate was incubated in the dark for 60 min at room temperature, after which its absorbance at 593 nm was read (EPOCH 2 microplate reader, BioTek^®^ Instruments, Winooski, VT, USA). The antioxidant activity of the samples was calculated by subtracting blanks to the samples, as well as the negative control, following which this value was normalized to that of positive control subtracting the negative control. The FRAP test was performed in triplicate and the results expressed as millimolar of iron II equivalents per milligram of seaweed extract (mM eq. Fe (II) mg^−1^ extract).

### 2.5. Antibacterial Activity

Relevant infectious agents of wounds were analyzed. The reference strains used in the antibacterial test were *Staphylococcus aureus* (ATCC 25923), *Pseudomonas aeruginosa* (ATCC 27853), *Escherichia coli* (ATCC 25922), *Klebsiella pneumoniae* (ATCC 11296), and *Proteus mirabilis* (ATCC 29906).

#### 2.5.1. Disc Diffusion Method

For the initial screening of all 15 fractions, the disc diffusion method was performed using *S. aureus* following the directrices of the Clinical and Laboratory Standard Institute (CLSI, 2014) reference documents M02 and M100-S24 [28]. Briefly, *S. aureus* was cultured in tryptone soya yeast extract agar (TSYEA, Sigma Aldrich, St. Louis, MO, USA), at 37 °C. Afterward, colonies of bacteria were resuspended in 0.85% saline solution, and turbidity was adjusted to 0.5 McFarland (approximately 1.5 × 10^8^ CFU·mL^−1^). Then, the inoculum was spread with a swab on Muller-Hinton agar (MHA, Sigma Aldrich, St. Louis, MO, USA) plates. Four paper discs (90 mm in diameter) containing 15 μL of each extract having a concentration between 50 and 100 mg·mL^−1^ were placed in the above MHA plate. As positive controls, discs containing ciprofloxacin, (CIP, 5 μg; Liofilchem, Italy), erythromycin (E 15 μg, Liofilchem, Italy) and gentamicin (CN 10 μg, Liofilchem, Italy) were used. One disc containing only DMSO was used as a negative control. After incubating for 18 h at 35 °C, the diameter (mm) of the inhibition zone was measured. The experiment was carried out three times and the mean values and standard deviation were presented.

#### 2.5.2. Microdilution Protocol

To characterize the antimicrobial potential of the selected extracts, the methodology applied was the microdilution procedure adapted from Clinical and Laboratory Standards Institute, using the reference document M7 (CLSI, 2015) [29] with minimal alterations. Briefly, bacterial colonies from solid medium growth of each of the five species selected were suspended in saline solution (0.85% NaCl) to a turbidity of 0.5 McFarland. This concentrated inoculum was diluted to 1 × 10^7^ CFU·mL^−1^ using a calibration curve of OD 625nm–CFU·mL^−1^ available for each bacterium. Of this diluted inoculum, 5 µL were used in each well so that a final concentration of 5 × 10^5^ CFU·mL^−1^ was achieved. The fractions to be tested were diluted in Muller-Hinton Broth II (MHB II, Sigma Aldrich, St. Louis, MO, USA) growth medium, in tubes, to a concentration of 1.5 mg·mL^−1^, originating the mix solutions. The mix solution, containing culture medium and extract, was filtered with a sterile syringe by sterile cellulose filters (0.2 μm) (VWR, Radnor, Pennsylvania, USA).

In this procedure, three different controls were used. The sterility control was the culture media Muller-Hinton Broth II. For the positive control of inhibition, ciprofloxacin (4 μg·mL^−1^) was utilized, and as negative controls (full growth or 0% inhibition), culture medium with DMSO (1.5% v/v). For each extract tested a respective control with mixed solution and culture medium was performed. The assay was performed in sterile round bottom 96-multiwell microplates (Thermo Scientific, Waltham, MA, USA), using a final volume in each well plate of 200 μL. After incubating for 20 h at 35 °C, optical density (OD) of 96-well plates was measured at 625 nm in a microplate reader (EPOCH 2, BioTek^®^ Instruments, Winooski, VT, USA). The experiment was carried out in triplicate.

### 2.6. Cellular Activity

#### 2.6.1. Cell Viability

A RAW 264.7 cell line (ATCC–TIB 71, mouse macrophages) was grown and maintained according to manufacturer instructions, using Dulbecco’s modified Eagle medium (DMEM, Sigma Aldrich, St. Louis, MO, USA), 10% Fetal Bovine Serum (FBS, Biowest, France), and a mixture of Nystatin (10 U·mL^−1^, Sigma Aldrich, St. Louis, MO, USA) and Kanamycin (100 mg·L^−1^, Sigma Aldrich, St. Louis, MO, USA). Microplates containing 5 × 10^4^ cells/well were incubated at 37 °C in 5% CO_2_ for 24 h. Cells were treated for 24 h with extracts (1:1 in DMEM, 10 % FBS). Each extract was used at 1.5 mg·mL^−1^ in phosphate-buffered saline (PBS, Lonza, Sweden). After the incubation period, the medium was removed by aspiration and washed with 100 µL of PBS. After that, 100 µL of DMEM with 5% FBS, without phenol red and supplemented with 3-(4, 5-dimethylthiazolyl-2)-2, 5 diphenyltetrazolium bromide (MTT, Sigma Aldrich, St. Louis, MO, USA) solution (0.5 mg·mL^−1^ in PBS) was added to each well to assess cell viability [30]. The microplates were incubated at 37 °C in 5 % CO_2_ for 4 h and then washed with PBS. After aspiration, 100 µL of DMSO was added and the microplates were agitated for a few minutes and kept in the absence of light until complete solubilization of formazan. The absorbance was read at 570 nm wavelength in a microplate spectrophotometer (EPOCH 2 microplate reader, BioTek^®^ Instruments, Winooski, VT, USA). PBS supplemented with the correspondent concentration of DMSO of each extract was used as a positive control and DMSO as a negative control. Each condition was tested using six technical replicates and three independent assays.

#### 2.6.2. Anti-Inflammatory Activity of Extracts (Nitric Oxide Production Inhibition)

A Griess diazotization reaction was used to measure the production of NO in RAW 264.7 cells according to Bahiense et al. [30] with slight modifications. Briefly, the microplates were seeded with 1 × 10^5^ cells/well and incubated at 37 °C in 5 % CO_2_ for 24 h. After that period, cells were treated with all concentrations (0.01, 0.001, and 0.0001 µg·mL^−1^) of extracts which cell viability was above 90% for 6 h, following the addition of a lipopolysaccharide (LPS, Sigma-Aldrich, St. Louis, MO, USA) solution at a final concentration of 1.5 µg·mL^−1^ for 22 h. Then, 150 µL of the supernatants of the cell culture was mixed with 50 µL of Griess reagent (Sigma-Aldrich, St. Louis, MO, USA) and incubated for 15 min at room temperature. The absorbance was measured at 540 nm using a microplate spectrophotometer (EPOCH 2 microplate reader, BioTek^®^ Instruments, Winooski, VT, USA) and three independent assays were performed. The inhibition of NO production rates (%) was calculated as percentages of control.

### 2.7. Chemical Characterization of Extracts

The chemical characterization performed in this study was intended for evaluating the presence of phlorotannins in two specific fractions, AQAE and wE100, since their extraction procedure and bioactivities analysis suggest the presence of these compounds.

#### 2.7.1. Total Phenolic Content (TPC) by Folin–Ciocalteu (FC) Assay

Total Phenolic Content was determined spectrophotometrically by using the Folin–Ciocalteu method, with some modifications [31]. Briefly, the working reagent (WR) was prepared daily by diluting one part of commercially available Folin–Ciocalteau reagent (2N with respect to the acid, Sigma-Aldrich, St. Louis, USA) in 14 parts of ultrapure water. TPC was determined by pipetting 10 µL of each standard/sample’s concentration per well or 10 µL of DMSO as control (eight wells/condition). In four of the eight wells, 200 µL of WR was added, and in the other four, 200 µL of 0.1 M HCl was added. The plate was incubated in the dark for 10 min, after which 40 µL of a solution of Na_2_CO_3_ 20% (w/v) was added to every well. Afterward, the plate was incubated for 45 min at room temperature, after which its absorbance at 765 nm was read (EPOCH 2 microplate reader, BioTek^®^ Instruments, Winooski, VT, USA). The TPC was expressed as milligrams of phloroglucinol equivalents (PGE) per milligram of seaweed extract (mg PGE·mg^−1^ extract). The assay was performed in triplicate.

#### 2.7.2. Nuclear Magnetic Resonance (NMR) Analysis

Samples were dissolved in 500 µL of DMSO-d_6_ (Sigma-Aldrich, St. Louis, MO, USA) and NMR experiments (^1^H and HSQC) were performed in a Bruker Avance 400 spectrometer (Bruker, Massachusetts, EUA) with a frequency of 400 MHz for ^1^H and 100 MHz for ^13^C. Chemical shifts were expressed in ppm and reported to the residual solvent signals.

#### 2.7.3. Fourier Transform Infrared (FTIR) Analysis

Fourier transform infrared (FTIR) spectroscopy with attenuated total reflectance (ATR) was used to evaluate the chemical composition of the two crude extracts chosen. FTIR analyses were carried out using an Alpha-P Bruker FTIR-ATR spectrometer (Bruker, Massachusetts, EUA), in the range of 4000–400 cm^−1^, at a 4 cm^−1^ resolution with 64 scans.

### 2.8. Statistical Analysis

The data are presented as mean ± SD of three independent experiments. SigmaPlot (V11.0) was used to perform the statistical analysis. All variables were tested for normality using the Shapiro–Wilk test. Afterwards, one-way analysis of variance (ANOVA) followed by Tukey test was used to compare the antioxidant activity between extracts in DPPH and FRAP assays, as well as antimicrobial activity, NO production and in quantification of phenolic content. A *p* value under 0.05 was considered statistically significant.

## 3. Results

The preliminary sequential extraction results (yields, antioxidant, and antibacterial activities) are presented in Supplementary Materials (S1), along with a brief discussion of the rationale for steps chosen to be included and excluded from the optimized sequential extraction. The results shown in the following sections are those for the optimized extraction.

### 3.1. Extraction Yield

The yield of the six fractions obtained in the optimized sequential extraction protocol are presented in Table 1.

The yield of extraction was higher in water-containing solvents (AQ—22.2% and E50—6.1%) and lower in the hexane fraction (0.5%). Ethanol extract yielded 2.0%, which was further divided in two fractions (E100H—0.9% and wE100—1.1%). The aqueous extract partitioned with ethyl acetate yielded the lowest value—0.3%.

### 3.2. Antioxidant Activity

**Figure 2:**
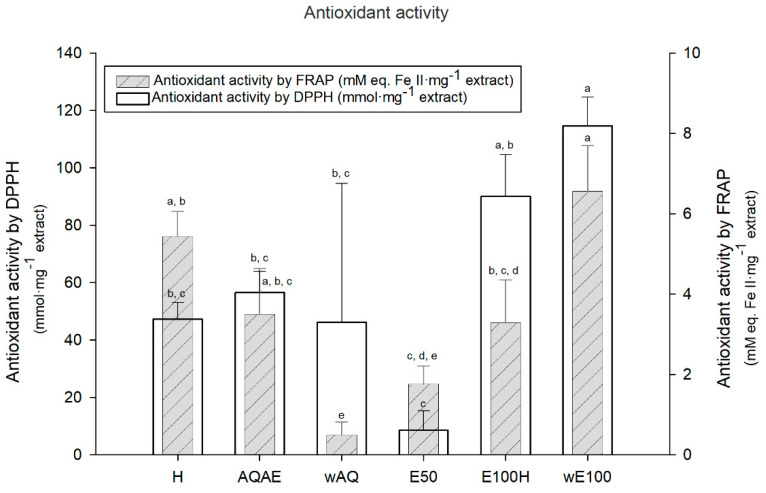
Antioxidant activity was evaluated by two different methods, Diphenyl-1-picrylhydrazyl (DPPH) and Ferric Reducing Antioxidant Power (FRAP) assay, of the six crude extracts of *Undaria pinnatifida* produced in optimized extraction. The results represent the mean ± SD of triplicate experiments. For each of the assays, the differences between extracts were evaluated using Tukey’s test. Bars with the same letters do not have statistically significant differences (*p* > 0.05).

The antioxidant activity displayed in the optimized extraction, evaluated by DPPH and FRAP, is presented in Figure 2. All extracts had measurable antioxidant activity by both methodologies. In DPPH, the highest radical scavenging activity was registered for both ethanol derived fractions, E100H and wE100. From highest to lowest, the extracts order was wE100 (114.61 ±10.04 mmol·mg^−1^ extract) > E100H (89.97 ± 14.62 mmol·mg^−1^ extract) > AQAE (56.54 ± 7.33 mmol·mg^−1^ extract) > H (47.24 ± 5.89 mmol·mg^−1^ extract) > wAQ (46.16 ± 48.34 mmol·mg^−1^ extract) > E50 (8.51 ± 6.80 mmol·mg^−1^ extract). In the case of FRAP, the highest values were obtained for wE100 and H. From highest to lowest, the extracts order was wE100 (6.56 ± 1.13 mM eq. Fe II·mg^−1^ extract) > H (5.44 ± 0.61 mM eq. Fe II·mg^−1^ extract) > AQAE (3.49 ± 1.15 mM eq. Fe II·mg^−1^ extract) > E100H (3.28 ± 1.07 mM eq. Fe II·mg^−1^ extract) > E50 (1.76 ± 0.45 mM eq. Fe II·mg^−1^ extract) > wAQ (0.49 ± 0.32 mM eq. Fe II·mg^−1^ extract).

### 3.3. Antimicrobial Activity

The results of antibacterial activity of the *U. pinnatifida* extracts against *S. aureus*, *P. aeruginosa*, *K. pneumoniae*, *E. coli,* and *P. mirabilis* are presented in Table 2 as mean of growth inhibition (% of the control, for extract at 1.5 mg·mL^−1^) and standard deviation. Ciprofloxacin control resulted in approximately 100% inhibition for all species. The extract AQAE was capable of totally inhibiting the growth of *S. aureus*. However, it did not inhibit the growth of the Gram-negative bacteria to a high degree (0–16.2% inhibition). Some extracts performed better overall than others: H and E100H had good intermediate inhibition results (19.46–76.11% and 0–78.06%, respectively); wE100 with medium values (19.49–44.79% inhibition); wAQ and E50 with lower values of inhibition (0–25.59% and 0–15.05%, respectively).

### 3.4. Anti-Inflammatory Activity (Inhibition of Nitric Oxide Production)

**Figure 3:**
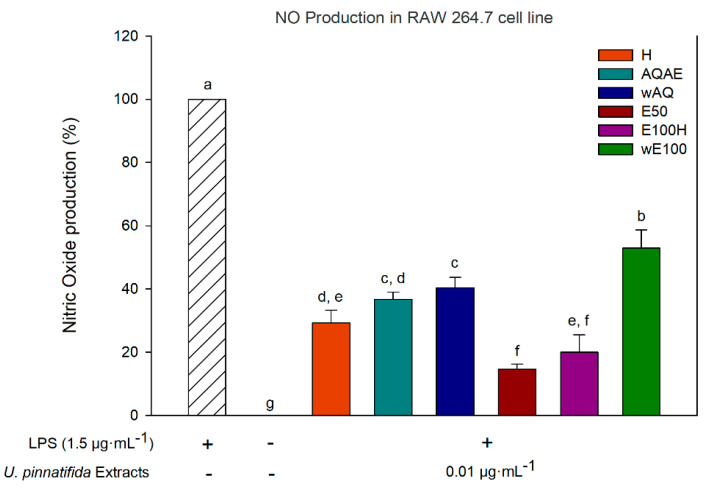
Nitric oxide production using the only noncytotoxic concentration (0.01 µg·mL^−1^) that showed significant differences with the lipopolysaccharide (LPS) treated group (*p* < 0.05) for each extract of *Undaria pinnatifida* in the evaluation of their anti-inflammatory potential, using a final concentration of 1.5 µg·mL^−1^ of LPS. The results represent the mean ± SD of triplicate experiments. Bars with the same letters do not have statistically significant differences (*p* > 0.05).

The inhibitory effect of the six fractions obtained in this study when used at a concentration of 0.01 µg·mL^−1^ (noncytotoxic concentration obtained from a dose–response curve with the cell line RAW 264.7) on the production of NO in lipopolysaccharide-stimulated RAW 264.7 is shown in Figure 3. In the presence of extracts, NO production was reduced to values between 14.6% and 52.9%. The most potent extract at reducing NO production was E50, followed by E100H, H, AQAE, wAQ, and finally wE100.

### 3.5. AQAE and wE100 Chemical Characterization

#### 3.5.1. Nuclear Magnetic Resonance (NMR)

**Figure 4:**
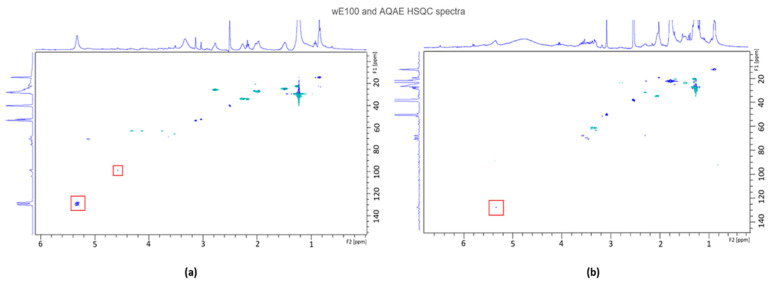
HSQC bidimensional NMR (proton and carbon-13) spectra of both extracts of *Undaria pinnatifida* selected for product development. (**a**) Spectrum of wE100 extract. (**b**) Spectrum of AQAE extract. Squares highlight possible phlorotannin peaks.

Bidimensional NMR spectra of proton and carbon-13 acquired by Heteronuclear Single Quantum Coherence (HSQC) experiments for wE100 and AQAE are presented in Figure 4.

In Figure 4a (the HSQC spectrum of wE100), three main regions of spots of correlation exist: the first, with δ(H) between 0.8 and 3 and δ(C) between 10 and 40; the second, with fainter signals, with δ(H) between 3 and 4.5 and δ(C) around 60; the third, a medium intensity spot (highlighted with a square) at δ(H) between 5.2 and 5.4 and δ(C) around 130. In Figure 4b (the HSQC spectrum of AQAE), two main regions of spots of correlation exist: the first, with δ(H) between 0.8 and 2.6 and δ(C) between 10 and 40; the second, with δ(H) between 3 and 3.8 and δ(C) between 45 and 70. However, in Figure 4b, a very faint signal (highlighted with a square) exists at δ(H) ≈ 5.4 and δ(C) ≈ 130.

#### 3.5.2. FTIR-ATR

**Figure 5:**
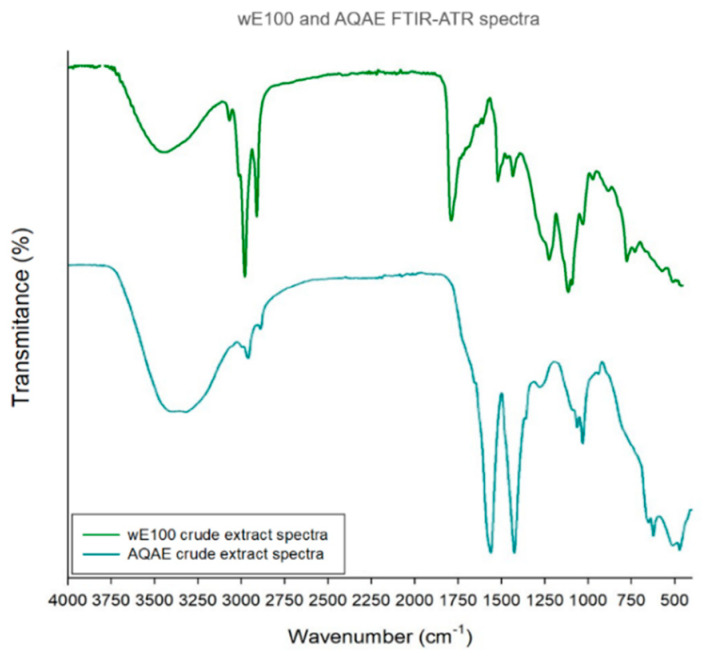
FTIR-ATR spectra of the dry wE100 (green line) and AQAE (cyan line) extracts of *Undaria pinnatifida*.

The FTIR-ATR spectrum was used to identify the functional groups of the active components present in wE100 and AQAE extract based on the band’s values (Figure 5). A more detailed description of the identified bands is present in Table 3.

#### 3.5.3. Total Phenolic Content (TPC) Quantification

To further corroborate the NMR and FTIR-ATR findings, AQAE and wE100 total phenolic content (TPC) was determined using the Folin–Ciocalteu assay and expressed in relation to phloroglucinol equivalents (PGE). The TPC values obtained were 0.24 ± 0.025 mg PGE·mg^−1^ extract for AQAE, and 0.48 ± 0.047 mg PGE·mg^−1^ extract for wE100. These values were statistically different (*p* < 0.05) using Tukey’s test.

## 4. Discussion

In this study, the optimization of a sequential SLE followed by LLE, using the least toxic solvents possible, in low volumes, with low energy input, was developed for the brown seaweed *U. pinnatifida*. Thus, an industry- and environmentally friendly approach was used to tentatively recover a fraction enriched in phlorotannins, while also obtaining other bioactive fractions. Data of the preliminary sequential extraction is presented in Supplementary Materials (S1), along with a brief discussion of the rationale for steps chosen to be included and excluded from the optimized sequential extraction. The optimized fractions were characterized concerning their bioactivities, and two of them—the most likely to contain the phlorotannins, given the extraction protocol and bioactivity results—were chemically characterized by NMR and FTIR to evaluate the presence of phlorotannins.

As common in several phlorotannins extractions, the current procedure started with a pretreatment of algal material, a defatting process using *n-hexane* in order to avoid the downstream coextraction of lipidic components and/or pigments [21]. The yield of extraction for hexane (0.5%) shows that the defatting step of this extraction successfully removed approximately half of the lipid matter of the initial biomass since it is reported that *Undaria pinnatifida* possesses approximately 1% dry weight in total lipids [38]. The nonextracted lipids are likely polar lipids, more soluble in solvents such as dichloromethane, that were purposefully not used in this study due to their incompatibility with the bio-extractive industry (due to health and environment hazards). Regarding water:acetic acid (99:1) (wAQ + AQAE), the total obtained yield (22.2%) is explained by the extraction of polysaccharides and proteins, elements with a high massic contribution and that are highly soluble in water. Additionally, with diminutive contribution to the mass of AQ extract, the presence of acetic acid at 1% (v/v) possibly breaks some linkages of polysaccharides with phlorotannins, which allows the water-soluble phlorotannins to be extracted. Following this rationale, the partition of AQ with ethyl acetate (originating AQAE) serves a tentative recovery of these phlorotannins, and this fraction obtained an expectable low yield (0.3%). In the washed AQ (wAQ), compounds as mannitol, laminarin, and alginate [39], as well as monosaccharides such as rhamnose, xylose, and fucose [40] are expected for this species. Transitioning into the ethanolic extracts, the 50% ethanol (E50) is the second-highest yield obtained, which is explained since the presence of 50% acidic water still extracts high polarity components such as monosaccharides and amino acids [40]. Presumably, the majority of phlorotannins coextractants were removed by these upstream extracts and fractions, thus downstream extracts are more simplified in compound classes, and thus, more enriched in phlorotannins as expected for E100. However, to assure that other classes of compounds (other than phlorotannins, namely the carotenoids and polar lipids) are removed, a liquid–liquid partition with hexane was performed, which extracted 0.9% of these lipophilic compounds. The fraction wE100 is thus the one with higher chances of having phlorotannins in the largest amount.

In the wound healing process, oxygen is crucial, by eliminating invasive organisms, synthesizing collagen, acting on angiogenesis, and reepithelization [19]. Nevertheless, overproduced reactive oxygen species (ROS) lead to a delay in the wound healing process. Antioxidants from seaweed’s complex extracts are thus promising tools for wound healing since they are capable of neutralizing ROS [18]. Two methods were used to evaluate the antioxidant activity in order to better comprehend the mechanisms of the antioxidative potential of these seaweed extracts, as different classes of molecules present antioxidant activity by different mechanisms [41]. Using the DPPH method (antioxidant capacity by radical scavenging), the highest activity is observed in the fraction wE100, 114.61 ± 10.04 mmol·mg^−1^ extract, significantly higher than most fractions (*p* < 0.05). E100H and AQAE follow after wE100 with 89.97± 14.62 mmol·mg^−1^ extract and 59.54 ± 7.33 mmol·mg^−1^ extract, respectively. For FRAP activity (antioxidant capacity by metal ion reduction), wE100 registered once more the highest value with 6.56 ± 1.13 mM eq. Fe II·mg^−1^ extract (significantly higher than most fractions (*p* < 0.05)), followed by H (5.44 ± 0.61 mM eq. Fe II·mg^−1^ extract), AQAE (3.49 ± 1.15 mM eq. Fe II·mg^−1^ extract), and E100H with 3.28 ± 1.07 mM eq. Fe II·mg^−1^ extract. These values are comparable to those of other ethanol extracts of brown seaweed, as demonstrated by Tenorio-Rodriguèz et al. [33], where ethanolic extracts from *Eisenia arborea* showed significantly higher mean FRAP (11.9 µM FeSO_4_ µg^−1^), followed by *Padina concrecens* (6.8 µM FeSO_4_ µg^−1^), and *Cystoseira osmundacea* (5.4 µM FeSO_4_ µg^−1^).

In addition to the wE100 fraction, the AQAE fraction also stands out in both assays. Analogous results were obtained in the extraction of phlorotannins from *Fucus vesiculosus* by Wang and collaborators [23], where among different polarity fractions, the phlorotannin-enriched ethyl acetate fraction possessed the highest DPPH scavenging activity and reducing power. The highest values obtained for wE100 corroborate the a priori assumption that this fraction would be the richest in antioxidant molecules, possibly phlorotannins, since these are known to be excellent antioxidants [42], and most soluble in alcohols. Other fractions also showed antioxidant activities. However, not expected to be due to the presence of phlorotannins, as is the case of fraction E100H. The high DPPH and FRAP activity is possibly related to the presence of remaining fucoxanthin [43] and other carotenoids. Additionally, lipophilic extracts may contain metabolites with medium polarity and soluble in both alcohol and hexane, such as diterpenes, sterols, and fatty acids. Such compounds are also known to present relevant bioactivities, including antioxidant [44,45,46,47]. Another possibility is the presence of volatile compounds, already reported to have great antioxidant activity [45].

The antimicrobial activity of the six extracts of *U. pinnatifida* was analyzed against five pathogenic bacteria, commonly found in the wound bed and hospital environment, by microdilution test and compared with the common antibiotic ciproxoflaxin (CP). The six extracts have shown antibacterial activity, especially against Gram-negatives, which are more difficult to combat with natural extracts (or even known antibiotics), due to their increased level of resistance mechanisms obtained from having an additional mutable outer membrane [48]. While for Gram-positive (*S. aureus*) AQAE fraction, hexane extract (H), and a fraction (E100H) are the most efficient, for Gram-negatives, the highest activity was found for E100H followed by wE100. wE100 registered the maximum activity against *P. aeruginosa* with a growth inhibition of 44.79%, which might be due to phlorotannins as reported by the work of Cox et al. [49], where ethanol extracts were the most effective against this bacteria, achieving 93.89% inhibition. The efficiency of wE100 against *E. coli* and *P. mirabilis,* with inhibitions of 37.62% and 31.56%, correspondingly, could also be partly due to these compounds. The results obtained for the inhibition of *K. pneumoniae* with wE100 are more distinct, only with 19.49%. Against *S. aureus,* the ethyl acetate fraction of the aqueous extract had the highest growth inhibition with 102.07%, having a spectrum of action as effective as that of the commercial antibiotic, at the tested concentration. On the other hand, leftovers derived from aqueous extract, wAQ, have registered inhibition rate < 30%, because of their content in non-bioactive coextractants such as sugars and polysaccharides. Overall, ethanol and hexane derived extracts revealed good antimicrobial activity for all pathogens with differences from the aqueous fractions (*p* < 0.05), which might be due to the presence of fucoxanthin or halogenated volatile organic compounds (HVOCs) [13,50,51]. The antimicrobial activity of E100H against *K. pneumoniae* and *P. mirabilis* also prompts further investigation of this fraction, since *K. pneumoniae* is one of the most reported pathogens concerning antibiotic resistance [52,53], and *P. mirabilis* is frequently found in the hospital environment and causes significant clinical infections.

In the early phases of wound healing, NO production is mediated by the inducible nitric oxide synthase (iNOS) and cyclooxygenase-2 (COX-2) expression [13], which are responsible for chronic inflammatory response, particularly in chronic wounds. Thus, compounds that can reduce the production of NO, e.g., by reducing the expression of iNOS and COX-2, or other inflammatory agents, are promising therapeutics in wound healing and related inflammatory diseases. Cells pretreated with *U. pinnatifida* extracts at 0.01 µg·mL^−1^ and stimulated with LPS for 22 h revealed a significant reduction of NO production when compared with control treatment (*p* < 0.05) (Figure 3). The reduction of such NO production was more evident for E50 and E100H extracts at a concentration of 0.01 µg·mL^−1^ with 85.37% and 80.01% reduction, respectively. For the same concentration, other extracts showed the capacity to reduce the production of NO to 47.12% as observed for wE100. E100H, as an oily fraction of the ethanol extract of *U. pinnatifida*, may contain fucosterol, eicosatetraenoic acid (EPA), and stearidonic acid (SA), already described to be present in this species, which possess anti-inflammatory effects [54]. Similar results for other brown seaweed species exist in the literature. For instance, hexane fraction from *Myagropsis myagroides* ethanolic extract had a significant anti-inflammatory activity in LPS-stimulated BV-2 microglia [55]. *Ecklonia cava* ethanol extract, containing 10.6% w/w of dieckol, a phlorotannin type, decreased by more than half NO in LPS-stimulated RAW 264.7 cells, when treated with 100 μg·mL^−1^ [56]. Notably, the results obtained for wE100 may be related to the potential content in phlorotannins. Oddly, higher amounts of extract did not result in higher anti-inflammatory activities (data not shown), which was also verified by Catarino et al. [57] with *Fucus vesiculosus* phlorotannin-enriched extracts. Contrarily, Dong et al. [58] found that higher doses of *U. pinnatifida* sporophyll extracts had a higher anti-inflammatory effect. Nonetheless, in this work, extracts show an effective reduction of NO in very low concentrations, showing their potential to be used as an anti-inflammatory. Generally, other reports have NO reduction using seaweed extracts concentrations around 2.5–500 µg·mL^−1^ [58,59,60,61].

In this study, both wE100 and AQAE did not go through extensive purification procedures since such methodologies impact negatively the scalability and cost effectiveness of the process. Thus, it is expected that both fractions still contain a mixture of compounds. As such, the techniques used (bidimensional NMR and FT-IR) should not reveal a clear spectrum of a known phlorotannin, but if they were in appreciable amount, there should be indicative signals of their presence. In the case of wE100′s HSQC, intense signals can be observed in the region of δ(H) = 1–2 ppm and δ(C) = 20–40 ppm, which might be indicative of alkane derivates (carotenoids), such as fucoxanthin, as verified by Kim et al. on the seaweed *Eisenia bicyclis* [62,63] or its derivative fucoxanthinol [64]. Additionally, signals at those regions suggest the presence of fucoidan, by characteristic carbon signals at 16.0–16.7 ppm associated with the CH_3_ group of α-L-fucopyranose units [65], facts that are also verified by ^1^H NMR, with signals at 1.32 and 1.26 ppm being assigned to C6 methyl protons of L-fucopyranose [66]. Narrow signals at δ(H) = 2.14–2.21 ppm and δ(C) = 20.7–21.2 ppm may arise from CH_3_ protons of O-acetyl groups, which can possibly be from the monomers of fucoidan found in *U. pinnatifida*, e.g., fucose [65,66]. Some residues of Laminaran ((1,3)-β-D-glucopyranose and (1,6)-β-D-glucopyranose residues) might be expected, since their signals are recorded at δ(H) 3–4 ppm and δ(C) = 60–70 ppm [67,68,69], and are slightly visible in the spectrum. As highlighted in Figure 4a, wE100 revealed an intense signal at δ(H) = 5–5.5 ppm and δ(C) = 125–135 ppm, which has been already associated with phlorotannins (ether linkage arylC-O-arylC, found in phlorethols, fuhalols, and fucophlorethols) in the literature [70,71,72,73,74]. Additionally, some signals have been found in the ^13^C NMR spectra at δ(C) = 95–105 ppm, which are also associated with phlorotannins (arylC-arylC phenyl linkage in fucols, and =C-methine carbon of the aromatic ring) [74], however with very faint correlation signal.

In the case of AQAE’s HSQC spectra, it did not show the signals characteristics of phlorotannins in the region previously mentioned. Rather, the presence of mannitol along with other sugars is likely considering the signals at δ(H) ~4 ppm [69]. Similarly, some alkane-type signals of high intensity were found, which could be a signal of the presence of pheophytin. Some studies reported anti-inflammatory and antitumoral activities of pheophytin *a* [70]. While NMR analysis is a good method for identifying and quantifying purified phlorotannins, it is only qualitative in complex mixtures, which is the case, thus, it is not possible to identify specific compounds from the phlorotannins class, but it allows to strongly support that they are present in wE100.

Figure 5 introduces the FTIR-ATR spectral analysis of the fractions wE100 (green line) and AQAE (cyan line), with a broad intense band in 3300 cm^−1^ region which can be assigned to hydroxyl O-H stretch in intermolecular bonded alcohols or even residual moisture. Such moiety can be found in carbohydrates and polyphenols, among other compounds [33]. The range between 2800 and 2970 cm^−1^ corresponds to C-H stretch vibrations, which indicate the presence of alkanes [33], which could be found in both extracts. AQAE fraction spectrum shows broadband at 1408 cm^−1^, corresponding to sulfate S=O stretching, while wE100 presents a band at 1168 cm^−1^, representative of sulfonic acid. These bands are most likely originated from sulfated residues of fucose from sulfated fucans of *U. pinnatifida* [66]. The band at 1733 cm^−1^, in wE100, can be attributed to C=O stretching vibration of O-acetyl groups, while, at the wavenumber of 1463 cm^−1^, a sharp band was found and attributed to the aromatic ring stretch (C=C). Normally, the wavenumber for this group is around 1450–1510 cm^−1^ [32,33], and in phlorotannins-enriched extracts, it has been found between 1450 and 1470 cm^−1^ [75]. However, Synytsya et al. attributed the band at 1455 cm^−1^ to vibration of CH_2_ to galactose and xylose and CH_3_ to fucose [66], showing some possible interference of fucoidans and laminarans, typical of *U. pinnatifida*.

In the region of 1058 and 1020 cm^−1^, the bands for wE100 and AQAE shown can indicate the presence of phlorethols/fuhalols with ether bridges. This type of linkage, as well as the number and arrangement of phenolic hydroxyl groups in their structure, has a deep influence on the antioxidant properties in Laminariales species, as *U. pinnatifida* [33]. However, other ether linkages such as glycosidic bonds (also expected to be found in these extracts, given the presence of sulfate/sulfonic acid functional groups, e.g., in oligosaccharides) may also vibrate in this frequency. Adding to this information, a little broad peak also is noticed at 720 cm^−1^ for wE100 extract, which relates to methine bending vibration in benzene derivatives—a characteristic of phlorotannins [32]. Together, these bands strongly suggest the presence of phlorotannins in wE100 extract.

In fact, the polyphenols quantification procedure used corroborated the NMR and FTIR results by showing the wE100 as the fraction with higher phenolic content (*p* < 0.05). The values observed, 0.24 ± 0.025 mg PGE·mg^−1^ extract for AQAE and 0.48 ± 0.047 mg PGE·mg^−1^ extract for wE100, are in accordance with others found in literature [76]. The use of ethyl acetate to enrich extracts with phlorotannins is a common procedure in their extraction, as reported by Catarino et al. [77], in which the ethyl fraction of *Fucus vesiculosus* achieved the maximum phenolic content among other fractions, with 17.1 ± 1.5 mg PGE·g^−1^. Additionally, in the study of Wang and colleagues [23], the ethyl fraction of *F. vesiculosus* also had the higher TPC value with 88.3 ± 2.2 g of PGEs/100 g of extract. In the present study, the ethanolic fraction was shown to be the phlorotannin-enriched fraction, as found in the Cho et al. [78] study, where it was estimated that ethanol extracts of *Sargassum siliquastrum* had a significantly higher phenolic content (127.4 mg GAE^−1^ extract) than water or chloroform extracts. Additionally, when ethanolic and 50% EtOH extracts of *Sargassum muticum*, obtained at three different temperatures were compared, the highest content of phenolics was detected in EtOH extracts, while the lowest was found in water extracts [79]. This way, the results presented here do not agree with Machu et al. [80] findings, that reported that water is the best solvent for phenolics extraction from *Eisenia bicyclis*, *Sargassum fusiforme*, *Saccharina japonica*, and *U. pinnatifida* in comparison to the aqueous MeOH and Acetone extracts, neither the results from Leyton et al. [75], where the optimal conditions for the extraction of phlorotannins were pretreatment with hexane, followed by extraction with water. Actually, phloroglucinol polymers amount was higher in ethanol extracts, when analyzed both for 2,4-dimethoxybenzaldehyde (DMBA) and Folin–Ciocolteu, for several brown seaweed, *U. pinnatifida* included [81]. Despite the amount of evidence pointing towards an enrichment in phlorotannins in the extract wE100, it is important to emphasize that wE100, alike all the other extracts produced, is still a complex mixture of compounds (liquid–liquid extraction reduces the complexity but leads to semi-crude extracts). For that reason, even if the composition was known for all extracts, an association between a given class of molecules and the observed bioactivities could not be performed. For that, separation of the components and assaying them in separate would be required. The only relation that can be established between composition of the extracts and bioactivity, in this study, is between the polyphenols content of AQAE and wE100 and their antioxidant activity since these variables have been extensively correlated in the literature. Previous reports suggest that antioxidant activities are related with phenolic content [23]. In the Tenorio-Rodriguèz et al. [33] study, the high phenolic content of *E. arborea*, *P. concrecens*, and *C. osmundacea* can explain their antioxidant activity, since polyphenol compounds contain reducing properties, such as hydrogen or electron donating agents, which contribute to free radical scavenging potential. Lopèz and collaborators [82] verified a possible association between the antioxidant potency and the TPC. Cho et al. [78] also suggest that the scavenging effect on DPPH radicals of wild plant extracts is closely related to the phenolic content, and the effect is different depending on the polyphenolic compounds contained in the extract. Correspondingly, the ethyl acetate fraction of *F. vesiculosus* obtained by Wang et al. possessed the highest TPC as well as the strongest DPPH scavenging activity and reducing power [23].

## 5. Conclusions

In this study, a biorefinery framework for producing phlorotannins-enriched extracts and other valuable extracts from *U. pinnatifida* was developed, using conventional methodologies such as SLE and LLE, under the auspices of a new circular economy era. This work also presents an overview of the available innovative solutions to obtain, explore, and exploit seaweed compounds to apply in the biomedical field, since, new wound healing products with antimicrobial and anti-inflammatory activity are needed.

The evaluated results demonstrated that wE100 fraction suit those requirements, and analytical characterization by NMR and FTIR-ATR, highly suggests the presence of phlorotannins, that are further corroborated with the measurement of phenolic content through the Folin–Ciocalteu assay. It is worth noting that this fraction had potential antioxidant activity by radical scavenging (DPPH) and metal ion reduction (FRAP), and was able to inhibit Gram-negative bacteria growth, including principal hospital infection and antibiotic-resistant bacteria such as *P. aeruginosa*. The in vitro results indicated that wE100 acted against LPS-induced inflammation damage to RAW 264.7 cells, by inhibiting around 47% of the NO production at a minimal concentration of 0.01 µg·mL^−1^. The remaining fractions also have potential activities to be applied in same or other sectoral niches, due to their overall bioactivities. Notwithstanding, the fractions remain very complex, and those overall activities may well result from a synergy of compounds. Thus, further work is required for a complete characterization of phlorotannins-enriched extracts by advanced chromatographic and mass spectrometric techniques, as well as additional anti-inflammatory mechanism characterization, such as inflammatory genes’ expression inhibition.

In summary, the wE100 has the potential to be a wound-healing agent. Future perspectives like the incorporation of this extract in smart wound dressings are routes of work that should be addressed and be pursued. Additionally, it provides a cost-effective reason for the removal of this invasive seaweed, contributing to marine ecosystem restoration, while maximizing the value of *U. pinnatifida* for societal commodities and improved health.

## Figures and Tables

**Table 1:** Yield of extraction (%) of the six fractions of *Undaria pinnatifida* obtained in the optimized extraction.

Optimized Extraction Outputs			
Extraction Solvent	Liquid–Liquid Solvent	Extract Name	Yield (%)
*n*-hexane		H	0.5
Water:acetic acid (99:1)	Ethyl acetate	AQAE	0.3
Water:acetic acid (99:1)		wAQ	21.9
Ethanol:water:acetic acid (50:49.5:0.5)		E50	6.1
Ethanol	n-hexane	E100H	0.9
Ethanol		wE100	1.1

**Table 2:** Antimicrobial activity of all the six extracts of *Undaria pinnatifida* at 1.5 mg·mL^−1^ by broth microdilution assay against *Staphylococcus aureus*, *Pseudomonas aeruginosa*, *Klebsiella pneumoniae*, *Escherichia coli*, and *Proteus mirabilis*. Inhibition control was obtained using ciprofloxacin (CP). The results represent the mean ± SD of triplicate experiments. For each of the species, the differences between extracts were evaluated using Tukey’s test. Values with the same superscript letters do not have statistically significant differences (*p* > 0.05).

Strains	Growth Inhibition (%) for Extracts at 1.5 mg·mL^−1^						
	CP	H	AQAE	wAQ	E50	E100H	wE100
*S. aureus*	100.34 ± 3.16 ^a, b^	76.11 ± 12.64 ^a, b, c, d^	102.07 ± 1,53 ^a^	0.0 ± 26.15 ^f^	15.05 ± 9.99 ^e, f^	78.06 ± 9.06 ^a, b, c^	43.29 ± 2.85 ^d, e^
*P. aeruginosa*	101.17 ± 0.74 ^a^	38.99 ± 23.73 ^b^	0.0 ± 6.81 ^d^	1.75 ± 4.23 ^c^	0.0 ± 5.77 ^d^	0.0 ± 0.0 ^d^	44.79 ± 8.12 ^b^
*K. pneumoniae*	100.85 ± 3.75 ^a^	19.46 ± 5.03 ^c, d^	1.46 ± 1.08 ^e^	11.26 ± 0.53 ^c, d, e^	0.0 ± 1.57 ^e^	34.25 ± 7.81 ^b^	19.49 ± 5.42 ^c^
*E. coli*	99.93 ± 0,097 ^a^	40.98 ± 3.73 ^c^	16.24 ± 3.73 ^d^	25.59 ± 1.41^d^	0.0 ± 2.56 ^e^	54.9 1± 4.80 ^b^	37.62 ± 4.44 ^c^
*P. mirabilis*	100.97 ± 0,95 ^a^	23.88 ± 3.24 ^c, d^	14.15 ± 3.83 ^d, e^	8.18 ± 1.71 ^e, f^	0.0 ± 3.45 ^f^	68.79 ± 6.95 ^b^	31.56 ± 1.04 ^c^

**Table 3:** Main bands identified in the FTIR-ATR spectra of the dry wE100 and AQAE extracts of *Undaria pinnatifida* and potential groups associated

WE100 and AQAE FTIR-ATR Spectra				
Extract	Main Bands			References
	Wavenumber (cm^−1^)	Bond/Vibration	Functional Group	
wE100	3366	O-H stretch	Hydroxyl	[32,33,34,35,36,37]
	2922	C-H stretch	Alkane	
	2852			
	1733	C=O stretch	Aldehyde and esther	
	1463	C-H bend/C-C stretch	Alkane/Aromatic ring	
	1168	S=O stretch	Sulfonic acid hydrate	
	1058	C-O-C stretch	Unknown ether	
	724	C-H bond	Aromatic ring	
AQAE	3273	O-H stretch	Hydroxyl	
	2933	C-H stretch	Alkane	
	2852			
	1542	N-H bend/C-N stretch	Protein amides	
	1408	S=O stretch	Sulfate	
	1020	C-O-C stretch	Unknown ether	

## Data Availability

Not applicable.

## Supplementary Materials

The following are available online at https://www.mdpi.com/2218-273X/11/3/461/s1, Figure S1. Flowchart of the initial sequential extraction on the left and the liquid-liquid extraction with the respective outputs on the right. Extraction conditions are in accordance with the textual description in Section 2.2. in the main document; Table S1. Extraction yield (%), mass (mg), and antioxidant activities evaluated by DPPH and FRAP of the crude extracts obtained from Undaria pinnatifida; Table S2. Antimicrobial activity of all Undaria pinnatifida extracts, and commercial antibiotics (E, CIP, and CN) against Staphylococcus aureus obtained from agar disc diffusion assay, when tested at different amounts.
