# Supplementary material for: A Biorefinery Approach to the Biomass of the Seaweed Undaria pinnatifida (Harvey Suringar, 1873): Obtaining Phlorotannins-Enriched Extracts for Wound Healing
Source: Biomolecules. 2021 Mar 19;11(3):461. doi: 10.3390/biom11030461 (PMC8003497; doi:10.3390/biom11030461)
Supplement: Supplementary file 1 [file biomolecules-11-00461-s001.pdf]

The supplementary material S1 presented here includes the data correspondent to the preliminary extraction and is intended to justify the exclusion of certain extraction/fractionation steps between the preliminary and the optimized extractions. Briefly:

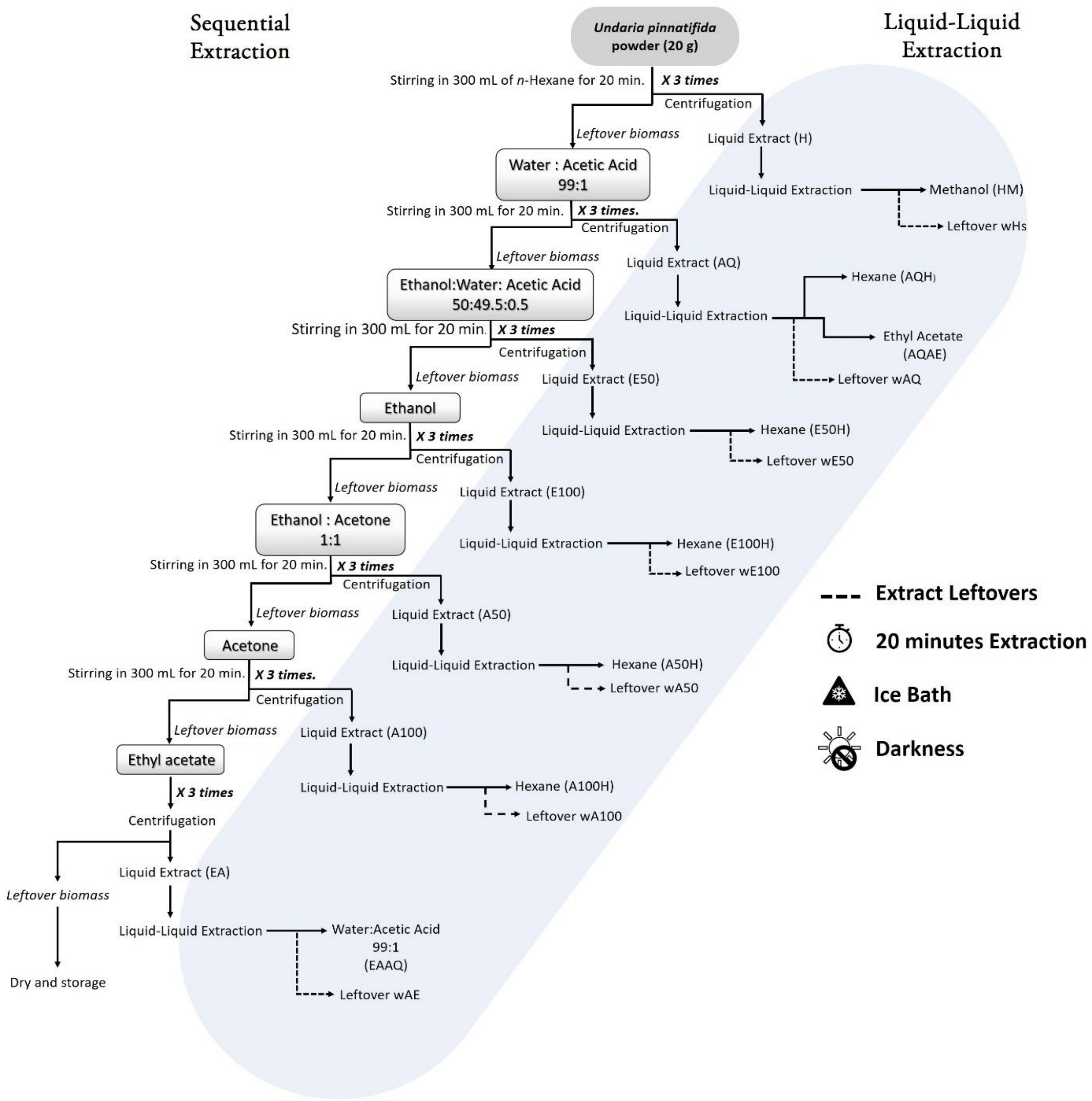

Figure S1. Flowchart of the initial sequential extraction on the left and the liquid-liquid extraction with the respective outputs on the right. Extraction conditions are in accordance with the textual description in 2.2. in the main document.

i) After ethanol was used, the extractions (ethanol:acetone (1:1), acetone and ethyl acetate) had very low yields, with medium results in antioxidant assays and no results in antimicrobial assays. Also, acetone and ethyl acetate are more expensive, less environment- and industry-friendly solvents. Altogether, these facts led to the exclusion of extractive steps posterior to ethanol (E100).

ii) The partition of the hexane extract (H) with methanol (and a few drops of water) did not result in the separation of the extract into two significant fractions. Rather, most of the original H remained in the wH; not only did it not result in significant mass separation (simplification of the fractions), it did also not result in the partitioning of antioxidant bioactivity all towards one of the two fractions. Therefore, despite the concentration of antibacterial compounds being much higher in HM than in H, the LLE step for H was dismissed since it did not likely contribute towards a phlorotannins-specific enrichment of a fraction.

iii) The partition of the water extract (AQ) with hexane was dismissed following the same rationale as the previous point. However, due to the very significant improvement in DPPH and antibacterial potentials of AQAE, combined with the theoretical fact that ethyl acetate could concentrate phlorotannins from AQ, the LLE with ethyl acetate was maintained in the optimized extraction.

iv) The partition of E50 into E50H and wE50 was dismissed for the same reasons as AQH partition was.

v) The LLE of ethanol extract (E100) with hexane significantly distributed the mass of the original extract into two fractions, thereby likely contributing to a simplification of its chemical composition. Also, E100 was the first extract of the pipeline that was almost certain to contain phlorotannins, and hexane as a “washing” solvent was almost certain not to remove them, so wE100 was a fraction that was worth further investigation, and therefore, LLE of E100 was maintained.

Table S1. Extraction yield (%), mass (mg), and antioxidant activities evaluated by DPPH and FRAP of the crude extracts obtained from *Undaria pinnatifida*.

Dry seaweed mass (g)	Extraction solvent	Liquid-Liquid solvent	Sample name	Mass (mg extract)	Yield (%)	DPPH activity (mmol.mg^{-1} extract)	FRAP activity (mM eq. Fe II.mg^{-1} extract)
25.017	*n*-hexane	Methanol	HM	9.3	0.04	Not detected	2.31 ± 0.01
	n-hexane		wH	103.9	0.42	Not detected	2.31 ± 0.06
	Water: acetic acid (99:1)	*n*-hexane	AQH	25.7	0.10	Not detected	1.78 ± 0.19
	Water: acetic acid (99:1)	Ethyl acetate	AQAE	28.1	0.11	68.68 ± 23.70	0.87 ± 0.24
	Water: acetic acid (99:1)		wAQ	6135.5	24.5	Not detected	0.48 ± 0.06
	Ethanol: water (1:1)	*n*-hexane	E50H	25.1	0.10	Not detected	4.43 ± 0.40
	Ethanol: water (1:1)		wE50	1201.2	4.80	25.86 ± 13.20	3.56 ± 0.06
	Ethanol	*n*-hexane[1]	E100H	299.9	1.20	71.31 ± 18.25	4.58 ± 1.93
	Ethanol		wE100	267.8	1.07	51.89 ± 11.72	5.47 ± 0.35
	Acetone	*n*-hexane[1]	A100H	5.4	0.02	Not detected	1.04 ± 0.05
	Acetone		wA100	6.6	0.03	251.04 ± 130.07	Not detected [*]
	Ethanol: acetone (1:1)	*n*-hexane[1]	A50H	51.5	0.21	112.18 ± 77.13	0.76 ± 0.09
	Ethanol: acetone (1:1)		wA50	24.5	0.10	194.39 ±19.61	0.28 ± 0.04
	Ethyl acetate	Water: acetic acid 99:1	AEAQ	78.7	0.31	30.87 ± 10.37	0.93 ± 0.09
	Ethyl acetate		wAE	18.5	0.07	42.96 ± 2.36	1.73 ± 0.060

[1] For these 3 extracts, *n-hexane* was chosen to remove the less polar compounds extracted. Since it was partially miscible with the extraction solvent, phase separation was forced with the addition of a few drops of *water*. [*] Not tested due to lack of extract

Table S2. Antimicrobial activity of all *Undaria pinnatifida* extracts, and commercial antibiotics (E, CIP, and CN) against *Staphylococcus aureus* obtained from agar disc diffusion assay, when tested at different amounts.

Control	Mean zone of inhibition (mm)	Type of inhibition	Amount (ug)
**Erythromycin (E)**	26.00 ± 1.00	H[1]	15
**Ciprofloxacin (CIP)**	24.00 ± 0.0	H	5
**Gentamicin (CN)**	21.33 ± 0.58	H	10
**DMSO**	0	ND[2]	10
Extract Name	Mean zone of inhibition (mm)	Type of inhibition	Extract amount (ug)
**HM**	11.3 ± 1.20	H	150
**AQAE**	9.3 ± 0.60	H	150
**wH**	12.0 ±.0.00	PH[3]	300
**AQH**	11.0 ± 0.00	PH	150
**E50H**	8.0 ± 0.00	PH	250
**wE50**	5.33 ± 4.62	PH	1500
**wAE**	2.67 ± 4.60	PH	225
**wAQ**	0.10 ± 0.00	MH[4]	375
**wA100**	0.10 ± 0.00	MH	200
**E100H**	0	ND	750
**wE100**	0	ND	750
**AEAQ**	0	ND	375
**A50H**	0	ND	300
**wA50**	0	ND	150
**A100H**	0	ND	75

[1] Halo [2] Not Detected [3] Pseudo Halo [4] Micro-Halo
